# Biodiversity as a firewall to engineered microbiomes for restoration and conservation

**DOI:** 10.1098/rsos.231526

**Published:** 2024-06-26

**Authors:** Victor Maull, Ricard Solé

**Affiliations:** ^1^ ICREA-Complex Systems Lab, UPF-PRBB, Dr. Aiguader 80, Barcelona 08003, Spain; ^2^ Institut de Biologia Evolutiva, CSIC-UPF, Passeig Maritim de la Barceloneta 37, Barcelona 08003, Spain; ^3^ Santa Fe Institute, 1399 Hyde Park Road, Santa Fe, NM 87501, USA

**Keywords:** ecological networks, bioengineering, synthetic biology, invasion dynamics

## Abstract

The possibility of abrupt transitions threatens to poise ecosystems into irreversibly degraded states. Synthetic biology has recently been proposed to prevent them from crossing tipping points. However, there is little understanding of the impact of such intervention on the resident communities. Can such modification have ‘unintended consequences’, such as loss of species? Here, we address this problem by using a mathematical model that allows us to simulate this intervention scenario explicitly. We show how the indirect effect of damping the decay of shared resources results in biodiversity increase, and last but not least, the successful incorporation of the synthetic within the ecological network and very small-positive changes in the population size of the resident community. Furthermore, extensions and implications for future restoration and terraformation strategies are discussed.

## 1. Introduction

Ecosystems are resilient entities capable of dealing with multiple sources of change, from environmental fluctuations to the undesirable outcomes of invader species. However, as with any other complex system involving positive feedback, they also involve breakpoints associated with changes in key parameters beyond given thresholds [1,2,3,4]. The increasing stressors associated with Anthropogenic drivers, from global warming to the increasing land use, are associated with a growing human population. These factors already affect extant populations globally, regarding biodiversity declines and defaunation. Because of the accelerated pace of change, rapid transitions are expected to occur in the following decades [5]. Such changes will profoundly affect ecosystem services on an unprecedented scale.

Different strategies have been proposed to protect ecological communities across scales, from conservation to restoration. A commonality in all these cases is biodiversity conservation: biodiversity is a firewall to invasion and an indicator of ecosystem health. However, the time window for response is shrinking rapidly, and other paths might need to be taken. These include the engineering of ecosystems that target their fragilities [6–8]. Such strategies involve different microbial engineering strategies, from probiotics to designing simple genetic circuits to perform specific functionalities. Can ecosystem bioengineering provide the right restoration effect while maintaining (or enhancing) biodiversity? What is the difference between using probiotics strains versus genetically modified cells? To answer these questions, dedicated efforts will be necessary to test them under controlled conditions to evaluate their effectiveness and safety. At this stage, models (both mathematical and computational) can provide great insight into potential implementations that fulfil our previous requirements. This article aims to provide such insight using a population dynamics approach.

**Figure 1.**
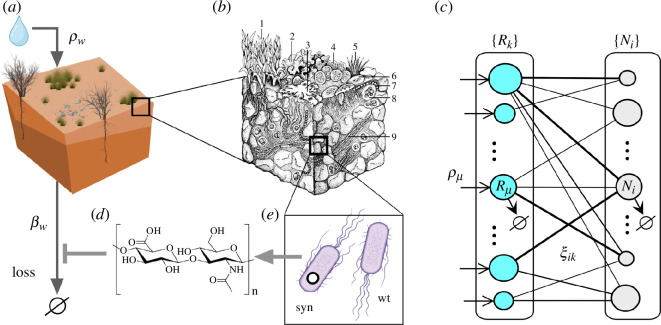
Targeting endangered ecosystems using synthetic microbiomes. Drylands (*a*) are a well-known example of ecosystems threatened by global warming and other anthropogenic drivers. A fundamental constraint here is water availability and loss. The soil crust associated with the upper layer of these systems ((*b*), adapted from [9]) involves a biodiverse community that is key to preserving their resilience and is also the target for potential engineering strategies. One possible path (*e*) is to engineer a synthetic strain (syn) from a resident, wild-type species (wt). The synthetic could produce an exopolymer (*d*) that could reduce water loss. One way of modelling the potential impact of such a strategy is the use of a resource–consumer network (*c*) where two sets R and C indicate available resources and those species exploiting them (weighted links indicate the strength of such interactions and the radius of the circles would scale as population size).

An example of such an engineering approach is shown in figure 1*a–d* for dryland ecosystems that are expected to experience significant decays or even collapse within this century owing to global warming [5,10,11,12]. Dryland soils (figure 1*b*) are known to include a rich community that includes the soil microbiome, which has been pointed out as a potential source of species candidates for engineering [8]. Microbiome diversity and soil organic carbon are crucial to preserving dryland systems, and both are major drivers of multifunctionality [13,14]. In the past, interventions have been used to restore degraded drylands [15,16]. Several studies involving inoculation of cyanobacteria have shown the potential for recovery, at least over some time [17,18]. However, a more directed approach using microbial bioengineering could provide a more efficient and durable effect [19,20]. One specific suggestion is summarized in figure 1. Here, we choose a resident species (wt in figure 1*e*) and obtain a synthetic strain (syn) that can secrete an exopolymer (d), which could act in reducing water loss. Such interventions can effectively push away the location of tipping points [21]. The role of exopolymers for desiccation tolerance is well-known in soil cyanobacteria [22,23] and exopolymers such as the external polysaccharide hyaluronic acid, have been synthetically produced by some microorganisms, for example *Escherichia coli* or *Bacillus subtilis* [24,25].

Interventions have also been suggested in other contexts associated with climate change and the microbiome [26,27]. This is the case for coral reefs, which are also threatened by global warming [28,29,30]. Here, too, health is correlated with microbiome composition and treatments against pathogens or microbial diversity imbalances have been approached with probiotics following dysbiosis [31], but engineered bacteria have also been pointed out as a promising alternative [32,33].

How reliable is the bioengineering alternative, and what can we expect from this kind of intervention? Are there unintended effects (such as extinction cascades) associated with such intervention? What kind of guidance can we get from a population-level study? Mathematical and computational models suggest that some well-defined ‘motifs’ can be implemented to modify the location and nature of tipping points [8,21,34–36]. However, these are low-dimensional models and cannot be used to test the potential effects of synthetic strains on a multispecies system.

In a previous work [37], we explored the problem of engineering a given species from a resident microbiome so that the modified strain would be reintroduced afterwards. This was the first-order approach to the more general problems outlined above. By design, we considered modifying the resident community, from which the synthetic strain is obtained, through a small change in one of its members. As a result, the synthetic organism would inherit most (if not all) of the wild-type links used as a target. This requirement is relevant because we start from a stable community with an associated ecological network. In this context, the introduced strain would be expected to be almost redundant and remain in place or replace the wild-type. Such scenarios were shown to occur under very general conditions within a multispecies competition model [37]. However, the engineered strain did not perform a specific functionality. In this article, we explore this situation using a resource–consumer multispecies model, where we target one species that will be modified to reduce the loss of one or more of the resources. As illustrated below, this approach yields biomass increase without incurring diversity losses, concurrently enhancing the critical resource’s abundance and the species that benefit from it.

## 2. Methods

### 2.1. Resource–consumer model

This study builds upon the established framework of consumer–resource models, explicitly drawing from the foundational work of MacArthur in the 1970s [38,39]. These models involve a multidimensional, nonlinear set of equations coupling a set of consumer populations, namely, $N_{i}$ where i=1,2,…⁢n , and a set of resources $\left\{R_{k}\right\}$ where k=1,2,…⁢m . The growth rates for each resource and species are represented by ρ∈(0,1) and η∈(0,1) , respectively. This class of model includes a set of equations for resource dynamics, namely,


(2.1) $$\frac{d\,R_{k}}{d\,t}=\Gamma_{k}\,\left(\left\{\rho_{i},\xi_{i\,j}\right\},𝐍,𝐑\right)-\beta_{k}\,R_{k}$$

and the corresponding set for consumers,


(2.2) $$\frac{dN_{i}}{dt}=\eta_{i}N_{i}\left(\sum_{k}^{m}\xi_{ik}R_{k}\right)-\sigma_{i}N_{i}+\epsilon .$$

The term $\Gamma_{k}\,\left(\left\{\rho_{i},\xi_{i\,j}\right\},𝐍,𝐑\right)$ indicates the specific way in which resources are generated and consumed. Here, the set $\left\{\rho_{i},\xi_{i\,j}\right\}$ indicates all the required (growth and interaction) parameters and 𝐍,𝐑 are the population vectors for consumers and resources. Each consumer has a growth rate $\eta_{i}$ and a small immigration rate. Both populations have linear decay terms with associated rates $\beta_{k},\sigma_{i}\in \left(0,1\right)$ .

Here, we consider a scenario where resources enter the system at some rate $\rho_{k}$ and are consumed following a mass action term:


(2.3) $$\Gamma_{k}(\{\rho_{i},\mu_{ij}\},N,R)=\rho_{k}-\left(\sum_{i}^{n}\xi_{ki}N_{i}\right)R_{k}.$$

In this article, we have slightly modified MacArthur’s model equations (2.1–2.3) to accommodate our synthetic intervention requirements. We propose a set of differential equations that govern the model, consisting of one equation for resource abundance and another for population density over time. While we keep the structure of the consumer equations, the new form of resource dynamics now reads:


(2.4) $$\frac{dR_{k}}{dt}=\rho_{k}-\left(\sum_{i}^{n}\xi_{ki}N_{i}\right)R_{k}-F(R_{k},N_{syn}).$$

In the proposed model, the terms of the resource–consumer matrix $\xi =\left(\xi_{i\,j}\right)$ come from a uniform distribution *U*[0,1], with a connectivity C=0.3 .

The species decay rate is represented by σ∈(0,1) and to prevent unrealistically low biomass, a small immigration factor of $\epsilon =10^{-2}$ is introduced. Resource decay is driven by resource uptake by the species, mediated by the ξ matrix and a decay rate function $F\,\left(R_{k},N_{s}\right)$ associated with the set of resources and the synthetic population $N_{s}$ :


(2.5) $$F(R_{k},N_{s})=\frac{\beta_{k}R_{k}}{1+\delta_{k\alpha }\left(\frac{N_{s}}{\lambda }\right)}.$$

The function $F\,\left(R_{k},N_{s}\right)$ introduces a nonlinear modulation of the resource degradation process (see figure 1*a–d*). Specifically, the presence of a synthetic species in the community triggers a damping effect on the degradation of a specific resource α . The maximum decay rate for a given resource k is represented by $\beta_{k}\in \left(0,1\right)$ , while the constant λ∈(0,2) represents the rate of inhibition of the degradation process owing to the presence of the synthetic strain, $N_{s}$ . Lastly, $\delta_{k\,\alpha }$ is the Dirac’s delta function, i.e. $\delta_{k\,\alpha }=1$ when k=α and zero otherwise. Therefore, the damping process will only affect the targeted resources. See table 1 for a quick view of the parameters and functions described in the model.

**Table 1.** Quick view of the functions and parameters defined in the resource–consumer model.

variable	description
$R_{k}$	resource concentration, where k=1,2,...m
$N_{i}$	consumer concentration, where i=1,2,...n
$\eta_{i}$	consumer growth rates
$\sigma_{i}$	consumer decay rates
ϵ	small immigration factor
$\rho_{k}$	resource generation rates
$\beta_{k}$	resource decay rates
λ	rate of inhibition of the degradation process owing to the presence of the synthetic strain
$\delta_{k\,\alpha }$	Dirac’s delta function, i.e. $\delta_{k\,\alpha }=1$ when k=α and zero otherwise
ξ	resource–consumer matrix

### 2.2. Synthetic invasion

The core idea is to numerically test the effect of a synthetic invader, under a fundamental control framework, if such an organism can influence the extant environment. We take as a basic control framework the ideas particularly outlined in [37]. The underlying idea, in contrast with standard microbial invasions [40,41], propose a designed invasion scenario that considers both cell-level features as well as higher-order phenomena connected with community diversity. In order to modify ecosystems by introducing new functions using synthetic biology, there are vital aspects worth implementing in the design process. Using an extant species (wild-type) as a chassis for the synthetic strain (that will be introduced) can provide control and effective colonization outcome. The synthetic, under this specific layout, will have the same or almost the same community interactions as the wild-type. Therefore, the spread potential will be constrained by its niche. At the same time, the chances of successful establishment can increase substantially.

Here, we use a resource–consumer model to make explicit the relationship between the biomass community and the abiomass resource environment. The species compete for the resource pool through a bipartite network ξ , as specified in equations 2.2 and 2.4). Thus, a synthetic species is deployed within a resident community ${𝒞}_{R}$ , creating a new, *synthetic* community ${𝒞}_{S}$ . Because of our designed intervention, the synthetic species will essentially share the same interactions and similar growth rates with the wild-type strain. The simplest formulation is to assume that $\xi_{s\,k}=\xi_{w\,k}$ , but it seems reasonable to consider that a genetic modification process on a given strain can potentially change the metabolic balances and, therefore, alter their R–C interactions. To incorporate these deviations, we randomly shift the matrix elements by adding a noise term, i.e. $\xi_{s\,k}=\xi_{w\,k}\pm \Delta \,\xi$ , with Δ⁢ξ∈(0,0.1) a random number with a uniform distribution. The synthetic species’ functional impact will occur on a randomly chosen resource $R_{\alpha }$ . As discussed above, this effect involves a dampening in the resource degradation rate, as specified in equation (2.5). Additionally, the engineered strain is likely to have a fitness different from the wild-type. It can have a direct positive feedback effect on metabolic performance, or conversely, it can imply a metabolic burden. To incorporate these two options, we modify the efficiencies using $\eta_{s}=\eta_{w}\pm 0.1\,\eta_{w}$ . The neutral scenario ( $\eta_{s}=\eta_{w}$ ) will also be considered.

The intervention approach thus starts with an m -resources, n -species ecosystem. Each population will start the simulation with a small initial value of $N_{i}\,\left(0\right)=0.01$ and an initial resource amount of $R_{k}\,\left(0\right)=0.01$ . The community state ${𝒞}_{R}$ is followed over a transient of $\tau_{1}$ time steps, ensuring that the system is stable. At that moment, the community is considered stable and is therefore invaded with an extra species, $N_{s}$ , which will be based on a randomly chosen wild-type $N_{w}$ . The chosen mould species must have a minimal population $N_{w}(\tau_{1})>0.2$ . We invade with a small inoculation of synthetic strain as $N_{s}\,\left(\tau_{1}\right)=0.01$ . The decision of a small initial inoculum is not arbitrary. Our approach is grounded in the idea of engineering microbiomes at a large scale. We advocate harnessing the inherent biological capability for exponential and unsupervised growth as a cost-effective scaling technology. Therefore, the potential to propagate arises from design.

It is important to note that the set of species that compose the ${𝒞}_{R}$ does not change in terms of diversity because there is an immigration factor of ϵ that prevents total extinctions. Once the ${𝒞}_{R}$ is stable, the invasion takes place, leading the community to the synthetic state ${𝒞}_{S}$ , after time $\tau_{2}$ time steps, until the system reaches stability again. The R–C interaction matrix initially is:


$${𝒳}_{R}=\left(\begin{array}{cccc} \xi_{1,1} & \xi_{1,2} & \cdots & \xi_{1,n}\\ \xi_{2,1} & \xi_{2,2} & \cdots & \xi_{2,n}\\ \vdots & \vdots & \ddots & \vdots \\ \xi_{m,1} & \xi_{m,2} & \cdots & \xi_{m,n} \end{array}\right)$$

and is replaced by the ‘synthetic’ R–C interaction matrix, namely:


$$X_{R}=\left(\begin{array}{cccc} \xi_{1,1} & \xi_{1,2} & \cdots & \xi_{1,n+1}\\ \xi_{2,1} & \xi_{2,2} & \cdots & \xi_{2,n+1}\\ \vdots & \vdots & \ddots & \vdots \\ \xi_{m,1} & \xi_{m,2} & \cdots & \xi_{m,n+1} \end{array}\right).$$

Alternatively, the control scenario where the invader is a random species has also been considered. Meaning by random that it does not come from a wild-type species present in the community. Therefore, such an introduced strain will have unknown random connections in the ξ matrix and a random η growth rate. We considered two interesting scenarios under this approach. First, the random invader does positively affect a randomly chosen resource, just as it is depicted in equation (2.5). This scenario mimics the introduction of an engineered microorganism (not based on an extant species) in order to perform the restoration task. Second, the random invader does not have a specific positive interaction with any resource.

A statistical analysis of the synthetic invasion impact is performed by determining the population change of the community from $\tau_{1}$ to $\tau_{2}$ . Single-event population changes and general biomass fold change have been studied in terms of statistical change of the extant species (excluding the wild-type and the synthetic strain). We generate 500 random stable resident communities ${𝒞}_{R}$ of m=10 resources and n=20 species. Each ${𝒞}_{R}$ community is randomly generated. Once each community achieves a stable state, it is subsequently invaded. To simulate the dynamics of the resource–consumer model, we used a fourth-order Runge–Kutta algorithm.

## 3. Results

In this section, we will study the general properties of the model, as described by equations (2.1–2.2). The first step is to consider the simplest model that captures the main features, but with additional assumptions to achieve a one-dimensional version. The general model is then considered using numerical experiments where the synthetic strain is introduced within a stable RC community, and the statistical patterns of population change are analysed and compared with those associated with a random invader performing the same functionality.

### 3.1. Two resources and two consumers

**Figure 2.**
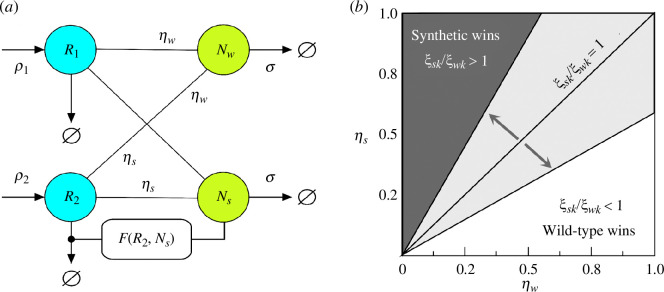
(*a*) The simplest case study of the general RC model would include two resources and two strains, one of which (the engineered one) reduces the loss of $R_{2}$ . The populations of the wild-type and synthetic strains are indicated by $N_{w}$ and $N_{s}$ , respectively. The synthetic would be obtained by a small engineering of the initial strain, which is indicated here as an open circle (indicating, for example, an engineered plasmid). Such a simplified system can be solved with four differential equations (3.1–3.4). The phase diagram in (*b*) summarizes the threshold conditions for the synthetic or the wild-type to grow. The success of one species over the other or the possibility of coexistence depends on their relative growth rates. In addition, their respective interaction matrix values are also part of the threshold condition if they are different. The diagonal lines represent (1) the simplest scenario, where $\xi_{w\,k}/\xi_{s\,k}=1$ divide the plane into two competitive exclusion areas divided by a coexistence line; and (2) when they are different $\xi_{w\,k}/\xi_{s\,k}\neq 1$ , the plane changes, widening or shrinking both areas (light grey).

Before we proceed to the general analysis of the multidimensional model, let us consider a simpler scenario that allows us to perform some analytic calculations and define the bounds required for a synthetic strain to persist. The simplified scenario is provided by a two-resource, two-species (wild-type and synthetic) system, as outlined in figure 2*a* diagram. As defined above, this engineered microorganism is designed to enhance the stability of one of the resources by reducing its loss. The basic diagram of this model is summarized in figure 2. In this section, we exploit the simplicity of this low-dimensional scenario along with an extra constraint that allows us to define the fundamental inequalities required for the synthetic strain to be successful.

For simplicity, we fix $\xi_{k}=1$ and assume a constant population constraint for the consumer populations, i.e. $N_{s}+N_{w}$ is a fixed quantity. This choice allows for reducing the initial system to a single-equation model. The equations now read


(3.1) $$\frac{dR_{1}}{dt}=\rho_{1}-(N_{w}+N_{s})R_{1}-\beta R_{1},$$

(3.2) $$\frac{dR_{2}}{dt}=\rho_{2}-(N_{w}+N_{s})R_{2}-\frac{\beta R_{2}}{1+\frac{N_{s}}{\lambda }},$$

for the resource dynamics and we also consider a modified set for the microbiome dynamics, namely:


(3.3) $$\frac{dN_{w}}{dt}=\eta_{w}N_{w}(R_{1}+R_{2})-\sigma N_{w}-N_{w}\Phi$$

and


(3.4) $$\frac{dN_{s}}{dt}=\eta_{s}N_{s}(R_{1}+R_{2})-\sigma N_{s}-N_{s}\Phi ,$$

where we indicate $\Phi =\Phi \,\left(N_{w},N_{s}\right)$ as the competition factor.

We can further reduce its dimensionality by assuming that the dynamics of the resources are fast, and thus we can consider that


(3.5) $$\left(\frac{d\,R_{1}}{d\,t}\right)=\left(\frac{d\,R_{2}}{d\,t}\right)\approx 0$$

and thus write the resource terms as a function of microbiome populations, i.e. after some algebra, we obtain


(3.6) $$R_{1}=\frac{\rho_{1}}{1+\beta }$$

for the first resource and


(3.7) $$R_{2}(N_{s})=\frac{\rho_{2}(\lambda +N_{s})}{(1+\beta )\lambda +N_{s}}.$$

Now, because of the assumption that the total population N remains the same, we consider for convenience that N=1 and, from the condition $d\,N/d\,t=d\,\left(N_{w}+N_{s}\right)/d\,t=0$ , it is possible to show that


(3.8) $$\Phi (N_{s},N_{w})=(\eta_{s}N_{s}+\eta_{w}N_{w})(R_{1}+R_{2})-\sigma .$$

Our main goal in this context is to guarantee that the synthetic strain can persist and define the conditions for such persistence. Using $N_{w}=1-N_{s}$ and the previous result, we have now, after rearranging some terms we obtain


(3.9) $$\frac{dN_{s}}{dt}=(R_{1}+R_{2})(\eta_{s}-\eta_{w})(1-N_{s})N_{s}.$$

Since $R_{1}+R_{2}>0$ , this single equation provides a threshold condition for the growth of the synthetic strain ( $dN_{s}/dt>0$ ), which in this case is simply


(3.10) $$\eta_{s}>\eta_{w}.$$

This result, under all the simplifications involved, suggests that a synthetic microorganism able to reduce the loss of a common good will likely establish itself, provided that its resource use efficiency is large enough. Specifically, the phase plane in figure 2*b* shows two well-defined areas above and below the $\xi_{w\,k}/\xi_{s\,k}=1$ line. Above the line, condition (3.10) is observed, thereby leading to a competitive exclusion in favour of our synthetic. Interestingly, at equal η , the condition for coexistence appears since both grow at equal speed until they reach the system capacity. Our derivation seems far from the multispecies problem, but—as shown below—this result is robust.

A more general case can be considered if deviations in the interaction matrix between the wild and synthetic components occur (as expected) when a genetic modification is introduced. Therefore, upgrading the coarse-grained model with $\xi_{wk}\neq \xi_{sk}$ states a scenario worth exploring. Therefore, the quartet of equations is the following:


(3.11) $$\frac{dR_{1}}{dt}=\rho_{1}-(\xi_{wk}N_{w}+\xi_{sk}N_{s})R_{1}-\beta R_{1},$$

(3.12) $$\frac{dR_{2}}{dt}=\rho_{2}-(\xi_{wk}N_{w}+\xi_{sk}N_{s})R_{2}-\frac{\beta R_{2}}{1+\frac{N_{s}}{\lambda }}.$$

Rearranging ψ=η⁢ξ :


(3.13) $$\frac{dN_{w}}{dt}=\psi_{wk}N_{w}(R_{1}+R_{2})-\sigma N_{w}-N_{w}\Phi (N_{w},N_{s}),$$

(3.14) $$\frac{dN_{s}}{dt}=\psi_{sk}N_{s}(R_{1}+R_{2})-\sigma N_{s}-N_{s}\Phi (N_{w},N_{s}).$$

We can reduce again the dimensionality by using the fast dynamics condition for resources equation (3.5) along with the constant population constraint. It can be shown that


(3.15) $$\Phi (N_{s},N_{w})=(\psi_{sk}N_{s}+\psi_{wk}N_{w})(R_{1}+R_{2})-\sigma .$$

And finally, rearranging the synthetic strain differential equation to the following expression. For the sake of clarity, we recover η⁢ξ=ψ :


(3.16) $$\frac{dN_{s}}{dt}=(R_{1}+R_{2})(\eta_{s}\xi_{sk}-\eta_{w}\xi_{wk})(1-N_{s})N_{s}.$$

Note that now $\left(R_{1}+R_{2}\right)$ , assuming again fast dynamics, reads


(3.17) $$\frac{\rho_{1}}{\beta -N_{s}(\xi_{wk}-\xi_{sk})+\xi_{wk}}+\frac{\rho_{2}}{\frac{\beta \lambda }{N_{s}+\lambda }-N_{s}(\xi_{wk}-\xi_{sk})+\xi_{wk}}.$$

Even though it changes with respect to equations (3.6–3.7); since populations are normalized to one, the sum is still positive for all scenarios. Overall, this provides a new threshold condition, namely,


(3.18) $$\eta_{s}>\left(\frac{\xi_{wk}}{\xi_{sk}}\right)\eta_{w}.$$

Here, the ξ term plays a multiplicative role with the intrinsic growth rate. Again, this result is reflected in the phase plane in figure 2*b*. Here, $\xi_{wk}/\xi_{sk}=1$ is a limited case of a broader picture. Therefore, when this ratio changes, it displaces the coexistence line further up if $\xi_{wk}>\xi_{sk}$ widens the failure spectrum of the wild-type, or the contrary effect is inverted. Extrapolating the pairwise dynamics to the multispecies problem becomes more challenging in this scenario owing to the dimensionality of the connectivity matrix. In general terms, it can be stated that the pairwise dynamics between the wild-type and the synthetic will fall on the same space of the possible: competitive exclusion, failure, and coexistence (when they are dynamically the same species), depending on the η⁢ξ relation. Notably, the coexistence domain expands compared to the previous scenario, driven by the increased possibilities arising from the combinatorics of η and ξ .

Finally, it can be noticed that this simplified coarse-graining neglects the effect of the small immigration factor ϵ present in the master equation (2.2). We avoided its addition for clarity since the dynamic attractors do not change. Nevertheless, since its presence affects the coexistence scenario, it is worth further exploration. Hence, ϵ is incorporated into the consumer equations, while the resource equations remain unaltered.


(3.19) $$\frac{dN_{w}}{dt}=\psi_{wk}N_{w}(R_{1}+R_{2})-\sigma N_{w}+\epsilon -N_{w}\Phi (N_{w},N_{s})$$

and


(3.20) $$\frac{dN_{s}}{dt}=\psi_{sk}N_{s}(R_{1}+R_{2})-\sigma N_{s}+\epsilon -N_{s}\Phi (N_{w},N_{s}).$$

We reduce dimensionality again by using the fast dynamics condition for resources (3.5) along with the constant population constraint. A competition term is defined now by


(3.21) $$\Phi (N_{s},N_{w})=(\psi_{sk}N_{s}+\psi_{wk}N_{w})(R_{1}+R_{2})+2\epsilon -\sigma .$$

Therefore, recovering η⁢ξ=ψ , the dynamics for the synthetic strain follows:


(3.22) $$\frac{dN_{s}}{dt}=(R_{1}+R_{2})(\eta_{s}\xi_{sk}-\eta_{w}\xi_{wk})(1-N_{s})N_{s}+\epsilon (1-2N_{s}).$$

The threshold condition remains for the first part of the equation:


(3.23) $$\eta_{s}>\left(\frac{\xi_{wk}}{\xi_{sk}}\right)\eta_{w}.$$

If equal, in the coexistence scenario, both species are only ruled by the small immigration term:


(3.24) $$\frac{dN}{dt}=\epsilon (1-2N).$$

Since the growth both species experience is ϵ plus a negative term dependent on each species’ abundance, it leads to a fixed point:


(3.25) $$N^{*}=\frac{1}{2}.$$

This result points towards a subtlety: the synthetic is expected to colonize half of the wild-type’s niche in the coexistence scenario owing to the predicted pairwise dynamics, no matter the initial abundances. If ϵ were too small, the predicted fixed point would be reached through a long transient.

### 3.2. Multiple species and resources

Consider now the full model, as described by the set of equations (2.2 *–* 2.4), along with the functional modulation of resources given by equation (2.5). This section aims to study, under general conditions, the effect of different strains (engineered or not) that invade an extant community. Two types of interventions are considered, namely, synthetic and random. We define as *synthetic* a genetically modified strain obtained from a wild-type species present in the resident community. The synthetic invaders can display different growth rate performances in relation to the wild-type organism. They can present a metabolic boost or burden or remain unchanged. On the contrary, we define those strains as *random*, genetically modified or not, and thus not based on any previous existing wild-type species of the extant community. Therefore, all the attributes are entirely aleatory.

**Figure 3.**
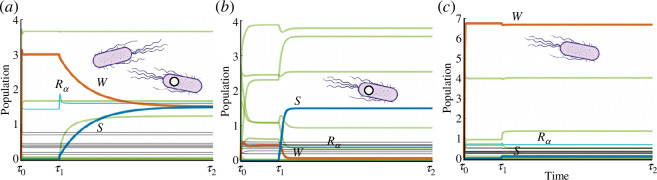
Population dynamics of engineered ecosystems where a synthetic strain is inoculated at $\tau_{1}$ . Thick orange, blue and green represent wild-type, synthetic and other species. The resources are represented in black, while the augmented resource ( $R_{\alpha }$ ) is indicated in light blue. In (*a*) , we have $\eta_{w\,t}=\eta_{s\,y\,n}$ which gives coexistence, see equations (3.10) and (3.25). In contrast, if $\eta_{s\,y\,n}=\eta_{w\,t}+0.1\,\eta_{w\,t}$ (*b*), a switch occurs, with the exclusion of the resident wild-type by the synthetic. Finally, in (*c*), the growth rate of the synthetic is 10% smaller than the wild-type owing to the extra metabolic load, leading to an extinction of the introduced strain after a transient time.


Different families of results are presented. We numerically generate the time series of the deployment scenarios (figure 3). Those time series highlight the pairwise dynamics between the wild-type and the synthetic and the possible impacts of a random invasion on the community. A statistical analysis is performed by measuring the frequency distributions of population shifts by analysing multiple runs of these time series. Specifically, the population change of the j th species after inoculation is computed as

(3.26) $$\Delta \,\left(N_{j}\right)=N_{j}\,\left(\tau_{1}\right)-N_{j}\,\left(\tau_{2}\right)$$

associated with the reconfiguration event post-invasion, accumulated through all the statistical trials. Here, $\tau_{1}$ is the last time step before the introduction of the synthetic, once the resident population has reached its stable state, and the final population is computed after $\tau_{2}$ steps (see §2).

In figure 3*a–c*, we display the three outcomes from our intervention scenario described above. As predicted by the two-species, two-resource scenario, the efficiency of the introduced strain largely determines the result of bioengineering. However, we can also evaluate its impact on community diversity: is the synthetic strain triggering community shifts? The answer seems to be negative, as illustrated by the time series. Here, we use thick orange and blue to represent wild-types and synthetic populations, respectively, while all other species abundances are shown in green. The resources are drawn in black, and the enhanced resource ( $R_{\alpha }$ ) in light blue. In figure 3*a*, we show a typical time series for the symmetric scenario ( $\eta_{wt}=\eta_{syn}$ ). As we appreciate, the two populations converge to the same population level, see equation (3.25). In contrast, if $\eta_{syn}=\eta_{wt}+0.1\eta_{wt}$ (figure 3*b*), a switch occurs. Finally, in figure 3*c*, the growth rate of the synthetic is 10% smaller than the wild-type owing to the extra metabolic load (i.e. $\eta_{s\,y\,n}=\eta_{w\,t}-0.1\,\eta_{w\,t}$ ). In this case, the synthetic fails to establish itself. In each run, a random wild-type species has been chosen from the pool with a population greater than $N_{w}§gt;0.2$ and the initial $N_{s}\,\left(\tau_{1}\right)=0.1$ . Here, the connectivity is C=0.3 . Note that in both scenarios in figure 3*b,c*, the species that end up failing are not completely washed out owing to the (small) immigration term ϵ in equation (2.2).

**Figure 4.**
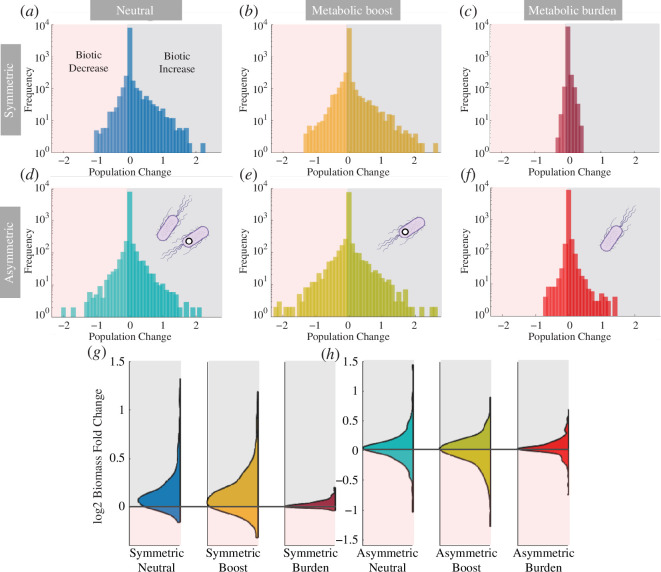
Statistical distributions of population changes for different scenarios, as defined by the population change $\Delta (N_{j})=N_{j}(\tau_{1})-N_{j}(\tau_{2})$ after the intervention (deployment of the synthetic) has been performed. Population expansion or shrink corresponds to $\Delta (N_{j})<0$ and $\Delta (N_{j})>0$ , respectively). The graph displays the accumulation of occurrences observed throughout 500 iterations. Graphs (*a–c*) correspond to the symmetric scenario where the synthetic strain replicates the wild-type in terms of the resource–consumer interaction matrix. Distribution asymmetries of positive skewness are observed. Conversely, graphs (*d–f*) represent the non-symmetric scenario where the R–C interaction matrix of the synthetic strain differs from that of the wild-type. These statistical distributions provide valuable insights into the dynamics of population changes and highlight the statistically null or positive effects of the synthetic intervention on the community. Furthermore, (*g*,*h*) stands as half-violin plots of the $\log_{2}$ total biomass fold change (see expression 32), comparing total biomass before ( $\tau_{1}$ ) and after the invasion ( $\tau_{2}$ ), averaged through 500 iterations. The areas in red define the biomass decrease as the $\log_{2}$ fold change is below 0. Inversely, the grey area stands as biomass increases.

What is the statistical nature of the previous patterns? Specifically, we want to analyse the expected impact of the synthetic ‘invader’ on the community structure. Exploring this question is strongly tied to the problem of whether such intervention can trigger ‘unintended consequences’. In figure 4, we display the distribution of changes in population density under the three different growth performances, under the two previous matrix choices. Each $N_{j}$ population experiences augmentations drops or no-change along 500 iterations of randomized initial conditions. All those changes are plotted and accumulated as a frequency of events. The population changes of the wild-type or the synthetic are neglected since the coarse-graining prediction holds, and it could mask the effect on the rest of the community. The plots are represented in a linear-log scale, thus helping to detect asymmetries. They also point to a first important result: the vast majority of population shifts are very small, thus indicating that the introduced strains have little impact at this level. In all plots, we have indicated the $\Delta (N_{j})<0$ domain in red. These are associated with a loss in species abundance and, therefore, a decrease in biomass. We highlighted the positive population changes in grey, marked as biomass increases. The fraction of events under this area measures the frequency of community changes that involve a marked reduction or augmentation of a component from time $\tau_{1}$ to $\tau_{2}$ .

Figure 4*a*–*c* corresponds to the (neutral) unchanged growth rate, metabolic boost and metabolic burden, respectively. All of them entail symmetric interactions between the wild-type and the synthetic ( $\xi_{sk}=\xi_{wk}$ ). Figure 4*d*–*f* corresponds to the same scenarios regarding the intrinsic growth rate, but the R–C interactions between the species are slightly randomized, $\xi_{sk}=\xi_{wk}\pm \Delta \xi$ , with Δ⁢ξ∈(0,0.1) . Interestingly, there is again a robust statistical skewness towards the positives in all scenarios, indicating a statistical biomass increase owing to the synthetic intervention. In other words, the introduced strain triggers more population improvements than population decreases, even though the skewness is more dramatic in the symmetric case (figure 4*a*–*c*).

The distribution of changes plotted in the histograms shows interesting information on single species drops during the invasion: if they are big or small, and if the positive ones are greater in number than the negative. Yet, a measure of the biomass fold change ℱ is needed to complement the impact of the invasion on the whole community. Figure 4*g,h* represents the $\log_{2}$ biomass fold change, calculated as:


(3.27) $$F(N_{i},\tau_{1},\tau_{2})=\log_{2}\left\{\frac{\sum_{j=1}^{n}N_{j}(\tau_{2})}{\sum_{j=1}^{n}N_{j}(\tau_{1})}\right\}.$$

The value of ℱ is estimated for 500 replicas of randomized ecosystems and represented in half-violin plots for each condition. Again, we support the same basic conclusions. Note again that the biomass increase or decrease associated with the wild-type or the synthetic is neglected since the coarse-graining prediction holds and could therefore mask the effect on the rest of the community.

**Figure 5.**
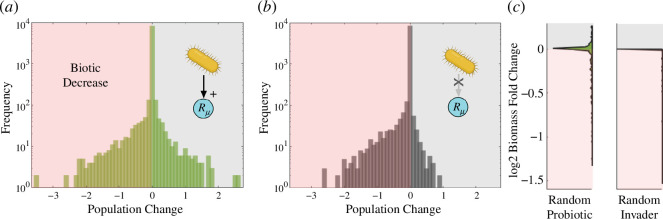
Statistical distributions of population changes for different scenarios of random invaders. Histograms are defined in figure 4. Here, the effects of random invaders are displayed, with (*a*) a positive effect over a given resource and (*b*) no effect on any resource. Distribution asymmetries are observed, highlighting a tendency for biomass decreases. Furthermore, (*c*) stands as a measure of the $\log_{2}$ total biomass fold change. The area of the half-violin plots here is small in comparison to figure 4 owing to the significant failure events.

Furthermore, the positive statistical biomass increase is highlighted when compared against a random invader, as shown in figure 5. Here, we find two scenarios that correspond to two relevant cases. Figure 5*a* corresponds to a random invader in all regards. However, the newcomer still possesses the ability to reduce a randomly chosen resource decay rate (here indicated by an arrow with a positive impact) and therefore represents a potentially engineered species, i.e. synthetic, yet not a copy of someone in the community. Second, in figure 5*b*, we display the results for a random invader without affecting resources. In both cases, the results show a dramatic difference. The skewness in the distribution of changes drives to the negatives, depicting a statistical biomass decrease resulting from the invasion. In figure 5*c*, we plot the $\log_{2}$ biomass fold change again and see a clear tendency towards biomass decrease. Furthermore, we can see that the overall impact on the extant community is limited, showing a very condensed distribution around 0.

This result points towards the fact that the random invasion is rarely successful. Nonetheless, a large tail of outliers spans towards biomass decreases, meaning that a fraction of events are highly harmful.

The comparison between the synthetic and random scenarios reveals important insights. In the first set of cases, it is observed that the synthetic invader consistently thrives. Whether the growth rate remains unchanged, it experiences a boost or a burden, the synthetic invader successfully establishes by coexisting with the wild-type or out-competing it. On the contrary, the random invader fails more frequently, along with poorer outcomes for the extant communities, as lessons from invasion ecology traditionally tell us [42]. The frequency distributions reveal high kurtosis values since they peak around zero (note that the frequency is on a logarithmic scale). The synthetic intervention scenarios exhibit right-skewed distributions, indicated by positive values, while the random scenarios are left-skewed.

**Figure 6.**
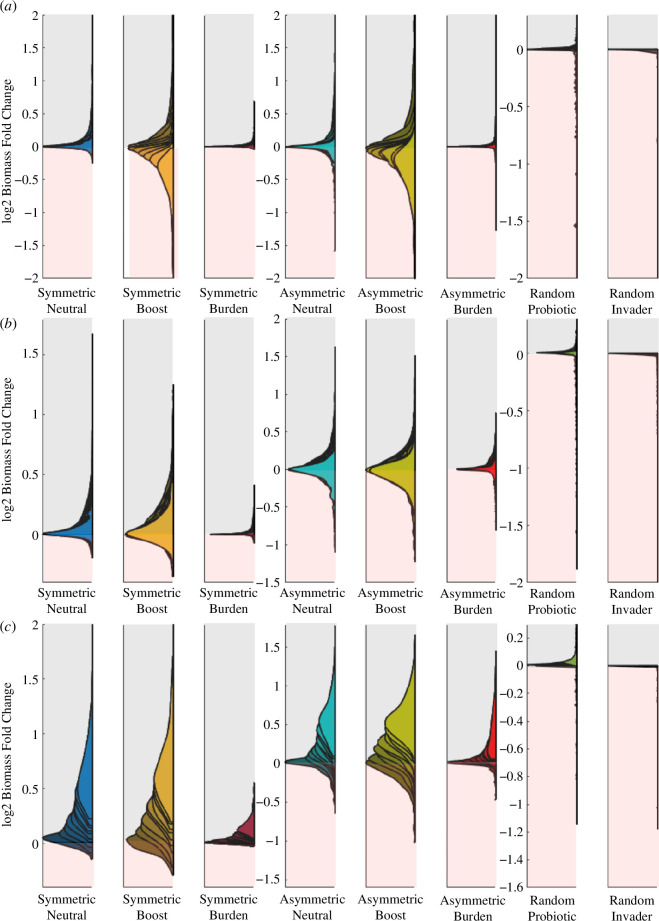
The effect of incorporating synthetic or random species in the community is plotted as the $\log_{2}$ fold change of the total biomass before ( $\tau_{1}$ ) and after the invasion ( $\tau_{2}$ ), for all case studies. Different additional conditions are plotted as overlapping half-violins: in (*a*), we plot fold change against different connectivity values in the ξ matrix, C∈(0.1,1) . In (*b*), we plot fold change against different λ values in equation (2.5), λ∈(0.1,1.9) . In (*c*), we plot fold change against the number of sensible resources to the positive effect of the synthetic, defined as the number of ones in the Dirac’s delta function in equation (2.5), from 1 to 10. The lighter the colour, the higher C , λ or the number of affected resources. The areas in red define the biomass decrease as the $\log_{2}$ fold change is below 0; inversely, the grey area stands as a biomass increase. Each half-violin distribution represents the average fold change in 500 experiments for each condition. The direct effects of both wild and synthetic types are not considered.

Notably, the symmetric scenarios (figure 4*a*,*b*) exhibit higher skewness values than the asymmetric scenarios (figure 4*d*,*e*). However, among the asymmetric ones, when there is a metabolic burden (figure 4*f*,*h*), we observe the highest positive skewness. This finding raises intriguing questions regarding the potential impact of positive and transient interventions. Can temporary synthetic invasions enhance the ecosystem at a broader scale and subsequently disappear, leaving behind a reorganized and healthier ecosystem? Furthermore, does this approach align with concerns regarding release control in natural environments? These problems are addressed again in §4.

Further numerical simulations were done to assess the robustness of the results when other parameter changes were considered, namely, (i) different connectivity values, (ii) different rates of inhibition of the degradation process ( λ ), and (iii) different numbers of affected resources ( $\delta_{k\,\alpha }$ ). Connectivity captures most of the system-level universal properties of ecosystem complexity [43] and checking how the results vary across provides a measure of its robustness. The inhibition rate is an interesting measure to survey since it can capture the variability of efficiencies of the synthetic interventions on the environment. Finally, testing the number of resources involved in the positive feedback can be a proxy for different bioengineering targets, thus understanding the benefit of widening the intervention window. In this context, engineering the production of an exopolymer (figure 1) that could reduce water loss would positively affect the availability of many potential resources.

The results are summarized in figure 6. Biomass fold changes in $\log_{2}$ , are plotted for all different growth performances, both in synthetic scenarios and random invasions. Overlapping half-violin plots stand as the statistical result for all new conditions ranges. Here, the lighter the colour, the higher the C , λ or $\delta_{k\alpha }$ values. In figure 6*a*, we display biomass fold change against different connectivity values. We see an overall reduction of the positive biomass increase, especially visible in the symmetric and asymmetric boost scenarios. As the network becomes more connected, the effect of the newcomer is amplified whether the positive effect it produces is still constrained to a single resource. The more connected an ecological network is, the harder it is for an invader to penetrate it [42], yet remember that the synthetic can shortcut this effect thanks to the pairing with the wild-type species. Within the range of low C that is expected in natural systems, skewness remains mainly positive in all scenarios. On the other hand, the random invader remains inefficient in most of the trials, yet the biomass decrease area in red widens if compared with figure 5*c*. In figure 6*b*, we show biomass fold change against λ values. We can appreciate that the larger the λ , the smaller the effect of $N_{S}$ on the targeted resource, reducing the skewness of the distribution. Finally, in figure 6*c*, we show the biomass fold change when the synthetic has the potential to affect from 1 to the totality of the resources, interestingly raising the median of the results towards a higher biomass increase. The negative effect of a random invader never decreased through any of the tested conditions, highlighting the differences between random invasions and our proposed synthetic ones.

**Figure 7.**
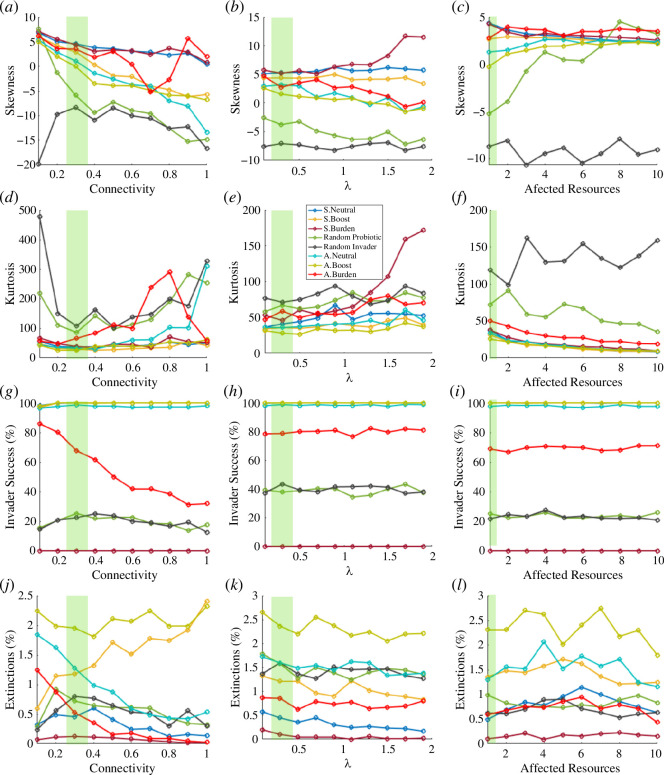
Line graphs allow us to see additional measures against interesting conditions such as connectivity, λ , and affected resources, already plotted in figure 6. In (*a–c*), we plotted the skewness of the statistical distributions of population changes. In (*d–f*), we plotted their kurtosis. Both measures are taken on the accumulation of occurrences observed throughout 500 iterations, see bar plots in figures 4 and 5. Differently, in (*g–i*), we plotted the percentage of invasion success ( $N_{S|I}>\epsilon$ ). Finally, in (*j–l*), we plotted the extinction percentage as a result of the invasion process ( $N_{j}\leq \epsilon$ ). Both measures, as the average throughout 500 iterations. Green areas stand for the scenarios shown in figures 4 and 5.

To gain further insight, in figure 7, the model is again tested using the whole parameter ranges of C , λ or $\delta_{k\alpha }$ . Figure 7*a–f* shows the skewness and kurtosis of the statistical population changes for all different conditions. Green areas stand for the previously shown conditions in figures 4*a–f* and 5*a,b*. We can clearly see how the skewness generally decreases as the connectivity or λ increases. Note that high kurtosis values mean high peaked distributions, thus the skewness results are more prone to be an effect of stochasticity. Interestingly, the skewness of the distributions does not seem to change in figure 7c. This is so because what is really changing here is the median, as shown in figure 6*c*. Regarding the random invader, here we can see a clear difference. When the random invader can affect more resources in the ecosystem, it can drive a positive impact more likely than its random counterpart. Figure 7*g–l* shows the ‘invasion’ success and extinction percentages. The ‘invasion’ success plots validate the coarse-graining predictions. The higher the connectivity, the closer the effect with the simplified scenario of two resources, two species. It can be clearly seen in the asymmetric burden scenario under increasing connectivity: neither λ nor $\delta_{k\alpha }$ affect the establishment of the newcomer. Finally, a note on the extinctions is needed, in figure 7*j*,*k*, the percentage of extinctions is very low, both owing to the little impact of the synthetic as a copy of a wild-type and the small initial inoculum of the random invaders. Nevertheless, interesting features can be seen. Extinctions decrease while the connectivity increases in all cases except in boost scenarios since the success of the species is granted by design and their effect is stronger. In the subsequent scenarios of λ and $\delta_{k\alpha }$ , extinction numbers seem to experience a slight reduction. In the first case, this is owing to the decrease in their effect on the environment, while in the second case, it is tied to the overall general benefit to the community.

All these results unequivocally illustrate the marked differences between the nature of random invasions and the proposed scenario of synthetic interventions. Furthermore, this study explores the nuances of effects when engineering microbiomes for restoration and conservation under the proposed framework, using the existing biodiversity (the natural network of interactions) as a firewall, both to protect and predict the effects on the extant communities and assure the success of the designed interventions.

## 4. Discussion

The utilization of microbial bioengineering may represent a crucial step in enhancing the resilience of ecosystems grappling with significant challenges posed by climate change. The pivotal question arises: can this strategy improve the reliability of ecosystems or will the introduction of engineered strains lead to undesirable effects at the community level, rendering them unsuitable for natural ecosystems? Despite ongoing debates regarding the use of engineered strains in conservation biology, there has been a notable absence of experimental tests or dedicated theoretical endeavours to address these questions. Consequently, the potential outcomes remain poorly understood. Moreover, a considerable gap exists between the predominant research approaches in synthetic biology, primarily on molecular and cellular scales, and ecosystem-level studies examining community dynamics across scales.

This study introduces a model of resource–consumer interactions to evaluate population-level changes resulting from the introduction of an engineered microbial strain. In contrast to a prior study [37], where the new strain merely competed with the resident community, our approach incorporates a specific functional enhancement of the resource pool. For a given resource, a synthetic strain, designed using a resident community member, reduces decay or spillover. The results indicate a generally positive impact, characterized by minor population changes, consistent with earlier findings. This suggests that contrary to concerns about unintended consequences, well-designed bioengineering can concurrently achieve two critical goals: modification as needed while preserving diversity. The strategy leverages emergent properties, ensuring that maintaining or increasing biomass and biodiversity also safeguard multifunctionality. In contrast to conventional ecological engineering [44], our approach targets the maintenance of high diversity as a specific engineering goal. This marks an essential conceptual innovation grounded in a systems-level engineering view, emphasizing emergent dynamics [8,45–47,48,49].

These results serve as a starting point for a more ambitious roadmap towards ecosystem modification. Numerous open problems remain, particularly on the theoretical side, where evolutionary, spatial, network dynamics and environmental architecture have been overlooked. Spatial dynamics, in particular, play a significant role as a biodiversity enhancer [50,51]. The interplay of resource and species dynamics across space and time, including stochastic dynamics, must be integrated into a richer context. In particular, network architecture is recognized for influencing the propagation of changes in trophic or mutualistic webs [52]. Similarly, while our bioengineering design has focused on a single species change, potential multispecies synthetic consortia may be relevant to achieving the desired effects, each targeting different resources. Experimental tests using micro- and mesocosm frameworks [53] are crucial to validate the theoretical ideas proposed in this study.

## Data Availability

This article has no additional data.

## References

[1] Scheffer M , Carpenter S , Foley JA , Folke C , Walker B . 2001 Catastrophic shifts in ecosystems. Nature New Biol. **413** , 591–596. (10.1038/35098000) 11595939

[2] Scheffer M . 2009 Critical transitions in nature and society. Princeton: U. Press. (10.1515/9781400833276). See https://www.degruyter.com/document/doi/10.1515/9781400833276/html.

[3] Lenton TM . 2011 Early warning of climate tipping points. Nat. Clim. Chang. **1** , 201–209. (10.1038/nclimate1143)

[4] Barnosky AD et al . 2012 Approaching a state shift in Earth’s biosphere. Nature New Biol. **486** , 52–58. (10.1038/nature11018) 22678279

[5] Solé R , Levin S . 2022 Ecological complexity and the biosphere: the next 30 years. Philos. Trans. R. Soc. B **377** , 20210376. (10.1098/rstb.2021.0376) PMC9234814 35757877

[6] de Lorenzo V , Marlière P , Solé R . 2016 Bioremediation at a global scale: from the test tube to planet Earth. Microb. Biotechnol. **9** , 618–625. (10.1111/1751-7915.12399) 27489146 PMC4993180

[7] Peñuelas J *et al* . 2013 Evidence of current impact of climate change on life: a walk from genes to the biosphere. Glob. Chang. Biol. **19** , 2303–2338. (10.1111/gcb.12143) 23505157

[8] Solé RV , Montañez R , Duran-Nebreda S . 2015 Synthetic circuit designs for earth terraformation. Biol. Direct **10** , 37, (10.1186/s13062-015-0064-7) 26187273 PMC4506446

[9] Belnap J , Lange OL . 2003 Biological soil crusts: structure, function, and management (10.1007/978-3-642-56475-8)

[10] Berdugo M , Kéfi S , Soliveres S , Maestre FT . 2017 Plant spatial patterns identify alternative ecosystem multifunctionality states in global drylands. Nat. Ecol. Evol. **1** , 3. (10.1038/s41559-016-0003) 28812618

[11] Berdugo M *et al* . 2020 Global ecosystem thresholds driven by aridity. Science **367** , 787–790. (10.1126/science.aay5958) 32054762

[12] Berdugo M , Vidiella B , Solé RV , Maestre FT . 2022 Ecological mechanisms underlying aridity thresholds in global drylands. Funct. Ecol. **36** , 4–23. (10.1111/1365-2435.13962)

[13] Delgado-Baquerizo M , Maestre FT , Reich PB , Jeffries TC , Gaitan JJ , Encinar D , Berdugo M , Campbell CD , Singh BK . 2016 Microbial diversity drives multifunctionality in terrestrial ecosystems. Nat. Commun. **7** , 10541. (10.1038/ncomms10541) 26817514 PMC4738359

[14] Delgado-Baquerizo M , Eldridge DJ , Ochoa V , Gozalo B , Singh BK , Maestre FT . 2017 Soil microbial communities drive the resistance of ecosystem multifunctionality to global change in drylands across the globe. Ecol. Lett. **20** , 1295–1305. (10.1111/ele.12826) 28921861

[15] Bowker MA . 2007 Biological soil crust rehabilitation in theory and practice: an underexploited opportunity. Res. Ecol. **15** , 13–23. (10.1111/j.1526-100X.2006.00185.x)

[16] Maestre FT , Martín N , Díez B , López-Poma R , Santos F , Luque I , Cortina J . 2006 Watering, fertilization, and slurry inoculation promote recovery of biological crust function in degraded soils. Microb. Ecol. **52** , 365–377. (10.1007/s00248-006-9017-0) 16710791

[17] Abed RMM , Dobretsov S , Sudesh K . 2009 Applications of cyanobacteria in biotechnology. J. Appl. Microbiol. **106** , 1–12. (10.1111/j.1365-2672.2008.03918.x) 19191979

[18] Sabarinatham KG , Gomathy M , Kumar DA , Kannan R , Aiyanathan EA . 2021 Cyanobacteria-mediated bioremediation of problem soils. microbial rejuvenation of polluted environment. In Microorganisms for sustainability, vol. 25. Singapore: Springer. (10.1007/978-981-15-7447-4)

[19] de Lorenzo V . 2022 Environmental Galenics: large-scale fortification of extant microbiomes with engineered bioremediation agents. Philos. Trans. R. Soc. B **377** , 20210395. (10.1098/rstb.2021.0395) PMC9234819 35757882

[20] Maestre FT , Solé R , Singh BK . 2017 Microbial biotechnology as a tool to restore degraded drylands. Microb. Biotechnol. **10** , 1250–1253. (10.1111/1751-7915.12832) 28834240 PMC5609258

[21] Solé RV , Montañez R , Duran-Nebreda S , Rodriguez-Amor D , Vidiella B , Sardanyés J . 2018 Population dynamics of synthetic terraformation motifs. R. Soc. Open Sci. **5** , 180121. (10.1098/rsos.180121) 30109068 PMC6083676

[22] Li P , Harding SE , Liu Z . 2001 Cyanobacterial exopolysaccharides: their nature and potential biotechnological applications. Biotechnol. Genet. Eng. Rev. **18** , 375–404. (10.1080/02648725.2001.10648020) 11530697

[23] Tamaru Y , Takani Y , Yoshida T , Sakamoto T . 2005 Crucial role of extracellular polysaccharides in desiccation and freezing tolerance in the terrestrial cyanobacterium Nostoc commune. Appl. Environ. Microbiol. **71** , 7327–7333. (10.1128/AEM.71.11.7327-7333.2005) 16269775 PMC1287664

[24] Widner B *et al* . 2005 Hyaluronic acid production in Bacillus subtilis. Appl. Environ. Microbiol. **71** , 3747–3752. (10.1128/AEM.71.7.3747-3752.2005) 16000785 PMC1168996

[25] Sze JH , Brownlie JC , Love CA . 2016 Biotechnological production of hyaluronic acid: a mini review. 3 Biotech **6** , 67. (10.1007/s13205-016-0379-9) PMC4754297 28330137

[26] Jansson JK , Hofmockel KS . 2020 Soil microbiomes and climate change. Nat. Rev. Microbiol. **18** , 35–46. (10.1038/s41579-019-0265-7) 31586158

[27] Jansson JK , Wu R . 2023 Soil viral diversity, ecology and climate change. Nat. Rev. Microbiol. **21** , 296–311. (10.1038/s41579-022-00811-z) 36352025

[28] Knowlton N . 1992 Thresholds and multiple stable states in coral reef community dynamics. Am. Zool. **32** , 674–682. (10.1093/icb/32.6.674)

[29] Knowlton N . 2001 The future of coral reefs. Proc. Natl Acad. Sci. USA **98** , 5419–5425. (10.1073/pnas.091092998) 11344288 PMC33228

[30] Lagerstrom KM , Vance S , Cornwell BH , Ruffley M , Bellagio T , Exposito-Alonso M , Palumbi SR , Hadly EA . 2022 From coral reefs to Joshua trees: what ecological interactions teach us about the adaptive capacity of biodiversity in the anthropocene. Philos. Trans. R. Soc. Lond. B Biol. Sci. **377** , 20210389. (10.1098/rstb.2021.0389) 35757872 PMC9234817

[31] Peixoto RS , Sweet M , Villela HDM , Cardoso P , Thomas T , Voolstra CR , Høj L , Bourne DG . 2021 Coral probiotics: premise, promise, prospects. Annu. Rev. Anim. Biosci. **9** , 265–288. (10.1146/annurev-animal-090120-115444) 33321044

[32] van Oppen MJH , Oliver JK , Putnam HM , Gates RD . 2015 Building coral reef resilience through assisted evolution. Proc. Natl Acad. Sci. USA **112** , 2307–2313. (10.1073/pnas.1422301112) 25646461 PMC4345611

[33] van Oppen MJH , Blackall LL . 2019 Coral microbiome dynamics, functions and design in a changing world. Nat. Rev. Microbiol. **17** , 557–567. (10.1038/s41579-019-0223-4) 31263246

[34] Solé R . 2015 Bioengineering the biosphere? Ecol. Compl. **22** , 40–49. (10.1016/j.ecocom.2015.01.005)

[35] Vidiella B , Sardanyés J , Solé R . 2018 Exploiting delayed transitions to sustain semiarid ecosystems after catastrophic shifts. J. R. Soc. Interface **15** , 20180083. (10.1098/rsif.2018.0083) 29925580 PMC6030637

[36] Vidiella B , Sardanyés J , Solé RV . 2020 Synthetic soil crusts against green-desert transitions: a spatial model. R. Soc. Open Sci. **7** , 200161. (10.1098/rsos.200161) 32968506 PMC7481726

[37] Maull V , Solé R . 2022 Network-level containment of single-species bioengineering. Philos. Trans. R. Soc. B **377** , 20210396. (10.1098/rstb.2021.0396) PMC9234816 35757875

[38] Chesson P . 1990 MacArthur’s consumer-resource model. Theor. Popul. Biol. **37** , 26–38. (10.1016/0040-5809(90)90025-Q)

[39] MacArthur R . 1970 Species packing and competitive equilibrium for many species. Theor. Popul. Biol. **1** , 1–11. (10.1016/0040-5809(70)90039-0) 5527624

[40] Mallon CA , Elsas J van , Salles JF . 2015 Microbial invasions: the process, patterns, and mechanisms. Trends Microbiol. **23** , 719–729. (10.1016/j.tim.2015.07.013) 26439296

[41] Vila JCC , Jones ML , Patel M , Bell T , Rosindell J . 2019 Uncovering the rules of microbial community invasions. Nat. Ecol. Evol. **3** , 1162–1171. (10.1038/s41559-019-0952-9) 31358951

[42] Case TJ . 1990 Invasion resistance arises in strongly interacting species-rich model competition communities. Proc. Natl Acad. Sci. USA **87** , 9610–9614. (10.1073/pnas.87.24.9610) 11607132 PMC55222

[43] Turnbull L *et al* . 2018 Connectivity and complex systems: learning from a multi-disciplinary perspective. Appl. Netw. Sci. **3** , 11. (10.1007/s41109-018-0067-2) 30839779 PMC6214298

[44] Odum HT , Odum B . 2003 Concepts and methods of ecological engineering. Ecol. Eng. **20** , 339–361. (10.1016/j.ecoleng.2003.08.008)

[45] Gorochowski TE *et al* . 2020 Toward engineering biosystems with emergent collective functions. Front. Bioeng. Biotechnol. **8** , 705. (10.3389/fbioe.2020.00705) 32671054 PMC7332988

[46] Holling CS . 1996 Engineering resilience versus ecological resilience. In Engineering within ecological constraints (ed. PC Schulze ), p. 32, vol. 31. Washington: National Academy Press.

[47] Krakauer DC . 2019 Emergent engineering: reframing the grand challenge for the 21st century. In Worlds hidden in plain sight, pp. 349–355. Santa Fe, New Mexico: SFI Press.

[48] Holling CS . 1973 Resilience and stability of ecological systems. Annu. Rev. Ecol. Syst. **4** , 1–23. (10.1146/annurev.es.04.110173.000245)

[49] Villa Martín P , Bonachela JA , Levin SA , Muñoz MA . 2015 Eluding catastrophic shifts. Proc. Natl Acad. Sci. USA **112** , E1828–E1836. (10.1073/pnas.1414708112) 25825772 PMC4403222

[50] Bascompte J , Solé RV . 1995 Rethinking complexity: modelling spatiotemporal dynamics in ecology. Trends Ecol. Evol. **10** , 361–366. (10.1016/S0169-5347(00)89134-X) 21237069

[51] Sole RV , Bascompte J , Valls J . 1992 Nonequilibrium dynamics in lattice ecosystems: chaotic stability and dissipative structures. Chaos **2** , 387–395. (10.1063/1.165881) 12779988

[52] Montoya JM , Pimm SL , Solé RV . 2006 Ecological networks and their fragility. Nature New Biol. **442** , 259–264. (10.1038/nature04927) 16855581

[53] Odum HT . 1996 Scales of ecological engineering. Ecol. Eng. **6** , 7–19. (10.1016/0925-8574(95)00049-6)

